# Occlusion of the Vagina

**Published:** 1881-12

**Authors:** Jno. G. Earnest

**Affiliations:** Atlanta, Ga.


### OCCLUSION OF THE VAGINA.

By JNO. G. EARNEST, M.D., Atlanta, Ga.

August 12, 1879. Mrs. C., æt 37; has borne three children—the last six years ago.

Five years since she had an attack of what appears, from her description, to have been vaginitis, complicated, probably, with inflammation of the cellular tissue of the pelvis, that confined her to bed for over two months. Since that time menstruation has been scant and painful, always accompanied at the beginning with nausea and cephalalgia. Sexual intercourse impossible. Catamenia exceedingly offensive, to the great annoyance of the patient. When seen, the menstrual period had just past; pulse, 120; skin hot; great tenderness over the ovarian region. Examination per vaginam discovered a cicatricial band stretched across the vagina, with a recession on each side. At the bottom of the recess on the right side was found an opening large enough to admit a Sims' sound. The withdrawal of the sound was followed by a discharge of half a teaspoonful of exceedingly offensive fluid, resembling a mixture of thin pus and blood.

The patient was placed on light diet, confined to the recumbent posture, given anodynes when necessary to relieve pain, and freely poulticed over the abdomen. At the end of ten days all tenderness had disappeared from the abdomen, and the pulse had gone down to eighty. The uterus was movable.

August 25. The cicatrix was divided with scissors, revealing the fact that it occupied about three-fourths of an inch longitudinally in the centre of the vagina, gradually sloping to a mere band of tissue at the sides. The vagina was found to be considerably narrowed by the cicatrix. The neck of the uterus was stripped of its epithelium, studded with granulations, and very much enlarged. The os was patulous and plugged with inspissated mucus. The after treatment consisted of lightly tamponing the vagina with cotton saturated with glycerine. This dressing was renewed daily, and the vagina washed out with tepid water, to which was added one per cent. of commercial carbolic acid. At the end of a fortnight all irritation had disappeared from the parts, and the wound fairly healed. The condition of the uterus was also found to be very much improved. The carbolic acid and tampon of cotton were left off, and the patient ordered to use tepid water injections twice daily.

September 29. The vagina was found to be contracted at the site of the cicatrix until there was little more than space enough left to admit the index finger. Under the circumstances it was deemed advisable to attempt the dilatation of the vagina to its original size. To accomplish this two pieces of sponge were cut into semi-cylindrical shape, so that when put together they would form a cylinder one and a half inches in length and two inches in diameter. A No. 8 bougie was fitted in the center (longitudinally,) between the pieces of sponge and then wrapped as an ordinary sponge tent. However, before wrapping, the sponge was moistened with a thin solution of gum arabic in water containing one per cent. of carbolic acid. When dry the cord and bougie were removed, and the tent covered with an ordinary rubber condom. A Sims' speculum was introduced and the lower portion of the perineum drawn back. The covering of the tent was oiled and the tent pushed through the contraction until it occupied the whole of the space covered by the cicatrix. With a Davidson syringe tepid water was then slowly injected through the opening left by the withdrawal of the bougie until the sponge was so far expanded as to retain its position and also a portion of the water above its insertion—this last precaution being required on account of all other sources of moisture necessary for the expansion of the sponge being cut off by the rubber covering. The patient then had a full dose of morphine and was put to bed for twenty-four hours. She was resting so comfortaby at the end of this time that the tent was allowed to remain sixteen hours longer. The tent having been made in sections was found to facilitate its removal. Its removal showed that we had gained a little ground; and the fact that it was only a little did not discourage us.

October 4. Another similar tent was introduced with scarcely any visible benefit after twenty-four hours.

October 8. Tent introduced and allowed to remain forty-six hours, when it was removed and the vagina syringed out with tepid water. As the menstrual period was now due no tent was introduced until October 14th, when all discharge had ceased. I may here state that her period was natural, free from pain, nausea or fever, and the discharge "no more offensive than from other women." This tent was allowed to remain in position forty-two hours. When removed the contracted collar was found to be markedly improved.

October 18. A tent was inserted and left in forty-six hours. As this was followed by slight tenderness of the upper portion of the vagina it was determined not to allow another to remain longer than twenty-four hours before removing and washing out the vagina.

October 23. All tenderness having disappeared another tent was introduced. This was followed by others every alternate day until November 7, when the vagina was found to be fully as large as natural at the former seat of contraction. The tissue of the cicatrix was markedly softened. All treatment was suspended except the daily use of warm water.

November 14. The patient stated that she believed that there was a return of the contraction. An examination seemed to substantiate her opinion, although it was not sufficiently well marked to be very apparent. Under the circumstances a tent was again introduced and allowed to remain twenty-four hours.

December 15. Another tent was introduced and left in thirty hours. After this she had no further treatment.

June 6, 1880. The patient reported that there had been no perceptible change in her condition. She has not since been heard from.

In conclusion we would call attention to the following points: To succeed in overcoming the contractions of cicatrical tissue in the vagina it is necessary that the sponge used should be fresh and highly elastic; that the tents when complete should be large enough to fill the opening to be dilated; that they should be made in sections to facilitate removal, as well as for economy. They should be covered with rubber to protect the mucous membrane from the action of the sponge. Finally, owing to the great power of resistance and the inveterate disposition to contraction, any or all of the means hitherto devised will sometimes fail. In any case it is necessary that the efforts at dilatation should be persisted in for a long while in order to secure the "alterative action" (whatever this is) upon the condensed tissue, brought about by the compression of the sponge; otherwise the contraction will certainly be reproduced.

